# Congenital Appendiceal Diverticulum: An Incidental Finding During an Appendectomy

**DOI:** 10.7759/cureus.14488

**Published:** 2021-04-14

**Authors:** Riley Gray, Roy Danks, Mackenzie Lesh, Alberto Diaz-Arias

**Affiliations:** 1 Medicine, A.T. Still University Kirksville College of Osteopathic Medicine, Kirksville, USA; 2 General Surgery, Northeast Regional Medical Center, Kirksville, USA; 3 Pathology, University of Missouri Hospital and Clinics, Columbia, USA

**Keywords:** diverticulosis, appendicitis, congenital diverticula, appendiceal diverticulosis

## Abstract

Diverticula are small outpouchings that form at weak points in the wall of the digestive tract. They are commonly found in the colon, and while they can occur anywhere in the digestive tract, occurrence in the appendix is rare. Here, we report the case of a young woman presenting with complaints of right lower quadrant abdominal pain. The patient’s physical examination, laboratory values, and computed tomography (CT) result supported the presumptive diagnosis of uncomplicated appendicitis. The patient underwent a laparoscopic appendectomy, where an appendiceal diverticulum was appreciated. A postoperative pathology report supported the diagnosis of a true appendiceal diverticulum. The patient’s symptoms improved postoperatively, and her recovery has been unremarkable. We conclude that appendiceal diverticula are often incidental findings that should be removed along with the appendix to reduce the risk of malignancy and perforation.

## Introduction

Diverticula are classified into two categories based on their histology. Acquired, or false, diverticula consist of mucosa and submucosa protruding through the muscularis, while congenital, or true, diverticula consist of all three layers protruding through a weak point of the bowel wall [1]. While diverticulosis is a relatively common pathology of the colon, the incidence of appendiceal diverticulosis is roughly 1%; and furthermore, the vast majority are acquired diverticula [1]. Appendiceal diverticula are often incidental findings not discovered until after surgery. However, when symptoms occur, they often mimic appendicitis [2,3]. When appendiceal diverticula are discovered, a prophylactic appendectomy is performed to eliminate the major risks of perforation and malignancy [3].

Here, we present a case of a congenital appendiceal diverticulum found incidentally during a laparoscopic appendectomy in a 19-year-old female who presented with right lower quadrant abdominal pain.

## Case presentation

A 19-year-old white female presented to the emergency department with the chief complaint of abdominal pain that began two days prior. The pain was initially periumbilical and localized to the right lower quadrant the night before. She described the pain as crampy and intermittent that did not radiate. She denied dysuria, fever, nausea, vomiting, melena, or hematochezia. Physical examination revealed a positive McBurney’s point and obturator sign. The remainder of her review of systems and physical examination was unremarkable. She had no relevant past medical or surgical history, and the only medications she took were oral contraceptives.

A complete metabolic panel, complete blood count, serum amylase, serum lipase, c-reactive protein, lactate level, urinalysis, and serum beta-human chorionic gonadotropin were ordered. The patient underwent a contrast-enhanced computed tomography (CT) of the abdomen and pelvis which found a thickened appendix with subtle periappendiceal soft tissue stranding, consistent with uncomplicated appendicitis.

A laparoscopic appendectomy was performed the same day, which revealed an inflamed and indurated appendix with a single diverticulum. The procedure was done in the supine position under general endotracheal anesthesia. The abdomen was clipped with hair, prepped with chlorhexidine, and draped in a sterile fashion. The incision sites were injected with a local anesthetic prior to incision. An incision was made below the umbilicus and the abdomen was insufflated with carbon dioxide to a pressure of 15 mm of mercury. A 5 mm port was placed below the umbilicus, and midline above the symphysis pubis, while a 12 mm port was placed in the left lower quadrant. The inflamed appendix was located, with no evidence of perforation on inspection, however, a solitary diverticulum was appreciated. Forceps were used to create a window in the mesoappendix, and an endoscopic linear cutting stapler was used to divide and staple the base of the appendix. The appendix was placed into an endoscopic retrieval bag, removed through the left lower quadrant port, and sent to pathology. The staple lines were irrigated and suctioned with 250 ml sterile saline, and hemostasis was confirmed. The left lower quadrant port site was closed with 0-Vicryl; then the abdomen decompressed while the trocars were removed. Skin incisions were closed with 4-0 Monocryl in addition to steri-strips and band-aids. The patient tolerated this procedure well and was taken to the postanesthetic care unit in stable and satisfactory condition.

**Figure 1:**
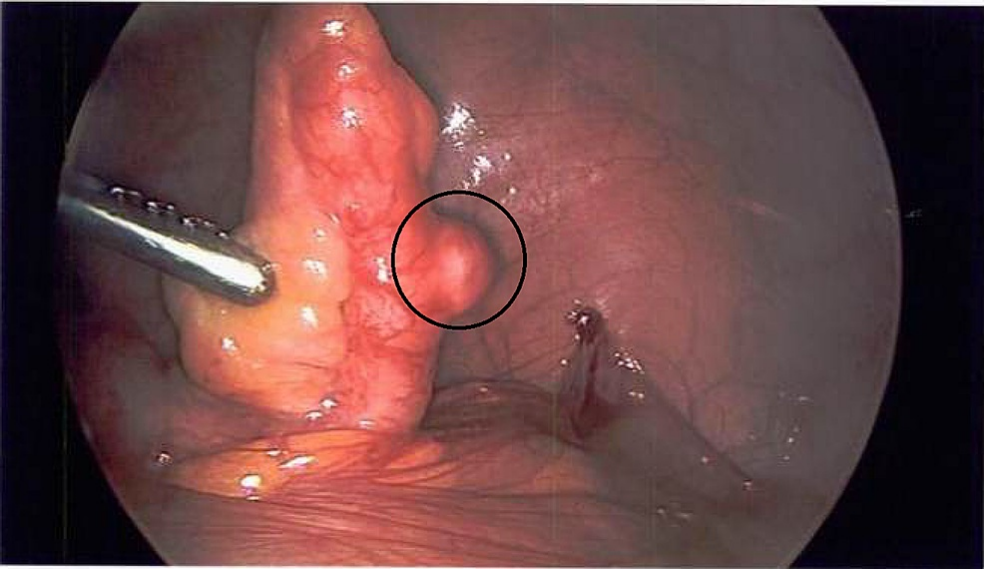
Intraoperative image of the appendix with the diverticulum circled.

**Figure 2:**
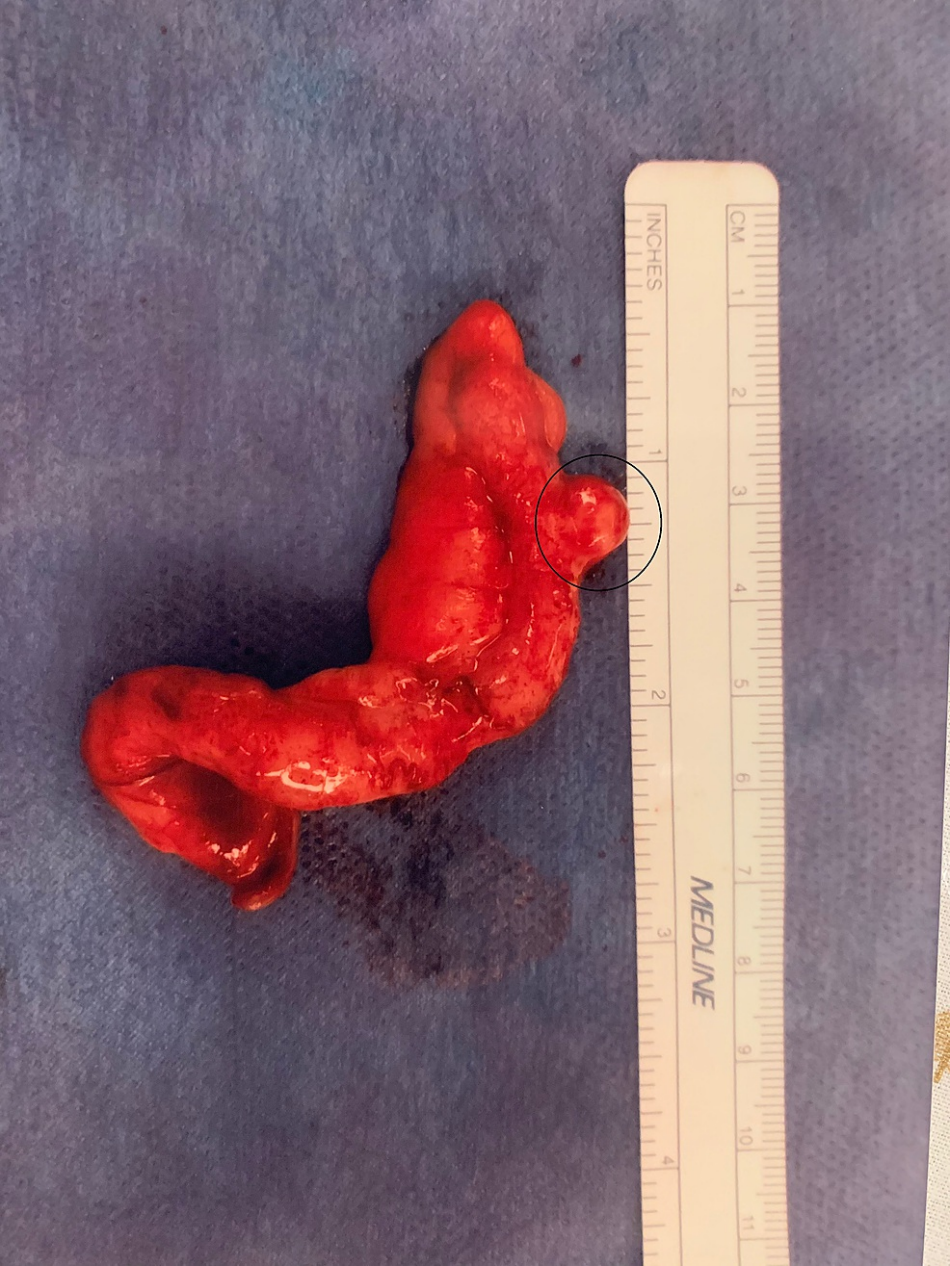
A gross image of the surgically removed appendix with the diverticulum circled.

**Figure 3:**
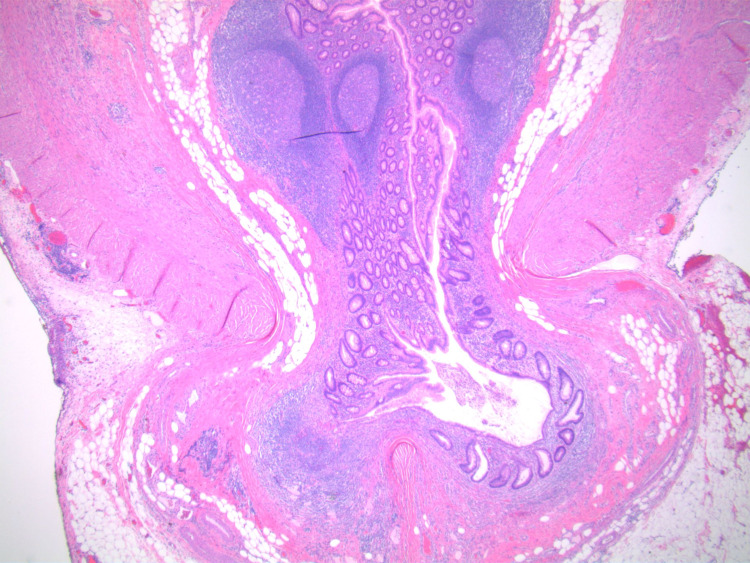
A cross-section of the diverticulum showing involvement of all three layers of the bowel wall.

**Figure 4:**
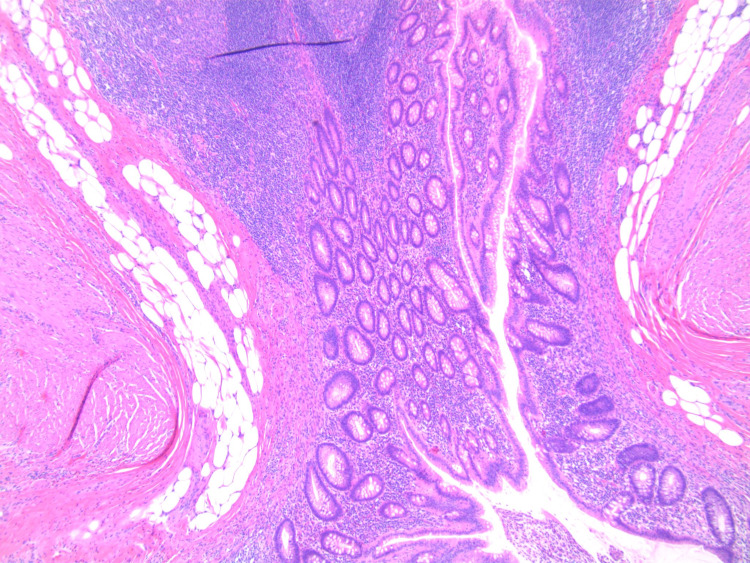
A magnified cross-section of the diverticulum showing involvement of all three layers of the bowel wall.

The pathology report confirmed the presence of acute and chronic appendicitis with associated diverticulosis and focal serositis. No dysplasia or malignancy was noted, and the diverticulum was confirmed to be a true diverticulum involving the mucosa, submucosa, muscularis, and serosa. The images of the gross appendix with the diverticulum, along with the images from the pathology report can be viewed in Figures 1-4.

## Discussion

Diverticulosis of the appendix is a rare and often incidental finding discovered during an appendectomy. The diverticula are typically asymptomatic, however, when symptoms do occur, they usually mimic appendicitis, including right lower quadrant abdominal pain, tenderness at McBurney’s point, fever, and leukocytosis. In contrast to appendicitis, appendiceal diverticulitis may present in middle age people with intermittent pain occurring up to two weeks before presentation [4]. Unsurprisingly, the treatment for both appendicitis and appendiceal diverticulosis is the surgical removal of the appendix. While this condition is considerably rare, possible risk factors for acquired appendiceal diverticula include male sex, age greater than 30 years, cystic fibrosis, and chronic appendicitis. Congenital appendiceal diverticula are exceedingly rare and are associated with group D chromosomal trisomy 13-15 [4].

Appendiceal diverticulitis can be divided into four subtypes based on the appearance of the diverticulum. Type 1 is a normal-appearing appendix with an acutely inflamed diverticulum, type 2 is an acutely inflamed diverticulum with surrounding appendicitis, type 3 is appendicitis with an uninvolved diverticulum, and type 4 is an incidental diverticulum with no appendicitis [4]. As shown in our case, a single diverticulum of the appendix can be an isolated finding not associated with diverticulitis of the colon.

When appendiceal diverticulosis is found, the appendix should be surgically removed and sent to pathology for a thorough histological evaluation because almost half of the cases with appendiceal diverticulosis also present with a neoplasm [5]. In addition to the increased risk of malignancy, it is important to correctly diagnose appendiceal diverticula because the risk of perforation is increased fourfold when compared to appendicitis, and it has been shown to be associated with pseudomyxoma peritonei. It is unclear whether the diverticulum increases the risk for pseudomyxoma peritonei, or if the diverticulum form from the increased pressure of mucin in the lumen [6]. Nonetheless, when appendiceal diverticula are suspected an appendectomy should be performed with careful consideration not to perforate the diverticulum and cause mucin to spill into the abdominal cavity. Other complications include GI tract hemorrhage, abscess formation, pelvic pseudocyst formation, and appendicovesical fistula formation [7]. Congenital diverticula are associated with fewer complications. This is likely due to the fact that the muscular layer is complete and able to protect the outer wall of the diverticulum [7].

If there is a high index of suspicion for appendiceal diverticulosis, it can be diagnosed by barium enema, right lower quadrant ultrasound, or CT [7,8]. Nonetheless, due to the rarity of this condition and the related symptomatology with acute diverticulitis, diagnosis is typically made post-operatively. However, it is important to note that there are imaging modalities available to make the diagnosis pre-operatively and possibly lead to a faster treatment to reduce the risk of perforation.

## Conclusions

This case of a congenital appendiceal diverticulum in a teenage female did not present as expected based on the literature, but it highlights an important diagnosis that is often overlooked. Diverticulosis of the appendix is difficult to diagnose on imaging and, like this case, is often diagnosed following surgery. Symptomatic appendiceal diverticulosis is managed with an appendectomy, and asymptomatic or incidental diverticulosis is treated the same way to decrease the risk of perforation or malignancy. Fatal complications can occur when left untreated or undiagnosed, therefore, it is important to have a high index of suspicion when a middle-aged male presents with intermittent right lower quadrant abdominal pain. It is also important to send the appendix to pathology to look for signs of malignancy or other underlying pathology.
